# Target Engagement Analysis and Link to Pharmacodynamic Endpoint for a Novel Class of CNS-penetrant and Efficacious p38α MAPK Inhibitors

**DOI:** 10.1007/s11481-014-9543-3

**Published:** 2014-05-01

**Authors:** Adam D. Bachstetter, D. Martin Watterson, Linda J. Van Eldik

**Affiliations:** 1 Sanders-Brown Center on Aging, University of Kentucky, 800 S. Limestone, Lexington, KY 40536 USA; 2 Department of Molecular Pharmacology and Biological Chemistry, Northwestern University Feinberg School of Medicine, Chicago, IL USA; 3 Department Anatomy and Neurobiology, University of Kentucky, Lexington, KY USA

**Keywords:** Mitogen-activated protein kinase, Drug discovery, Neurodegeneration, Signal transduction, Cytokine, Microglia

## Abstract

The protein kinase, p38α MAPK, is a key intracellular transducer of stressor-induced neuroinflammatory responses and, as such, is of high interest as a potential therapeutic target. We recently reported the synthesis and evaluation of first-in-class CNS-penetrant and highly specific p38 MAPK inhibitors that avoid target crossover issues seen in popular small molecule p38 MAPK inhibitors used in hundreds of previous reports. The novel p38 MAPK inhibitors, represented in this study by MW181, are efficacious in vivo. Pharmacodynamic actions include attenuation of stressor-induced increases in brain proinflammatory cytokine levels. We report here more detailed analyses of MW181 target engagement and specific linkage to the downstream increase in glia proinflammatory cytokine production. In vivo validation included demonstration that oral administration of MW181 suppresses lipopolysaccharide-induced increases in mouse brain IL-1β, TNFα, IL-6, IL-10, and CXCL1 but not in a drug-resistant p38α MAPK mutant mouse.

## Introduction

Neuroinflammation is a complex process that can be profoundly influenced by the cellular and environmental context, disease stage, and inciting stimuli. Microglia, as the resident tissue macrophage, are the archetypal cell in the CNS neuroinflammatory response. In addition, astrocytes, along with microglia, represent the resident cells in the nervous system responsible for neuroinflammation. In response to injury, infection, or other disturbances, microglia and astrocytes activate a patterned response to defend against and isolate inducing stimuli, which is followed by healing, repair, and resolution of the neuroinflammation. The reactive response of glia is fundamental for CNS homeostasis. However, the reactive glia response is context specific and highly variable, which can cause beneficial and/or detrimental forms of neuroinflammation (for a review of reactive gliosis see: Burda and Sofroniew 2014).

Microglia provide elegant examples of how a reactive glia response can be beneficial or detrimental. Microglia respond to a variety of pathological stimuli or other danger signals by turning on classical immune effector functions, characterized by the up-regulation of a battery of proinflammatory cytokines and chemokines, as well as oxidative and nitrosative stress molecules. These responses help to orchestrate and amplify beneficial repair responses that are vital to host defense against danger signals, and allow inactivation and/or phagocytosis of the pathogen or activating stimuli. However, these same microglial proinflammatory responses can contribute to downstream neuronal damage if not contained or attenuated in appropriate time windows after injury. For a microglia response to be beneficial, a careful balance must be maintained between protective/reparative and deleterious microglial activation. Moreover, a protective response in one context, such as a spinal cord injury, could be detrimental in a diffuse brain injury, or irrelevant in a degenerative disease. Overall, if neuroinflammation is inefficient, excessive, or prolonged, the delicate neuroinflammatory balance will become disrupted resulting in tissue damage, including neurodegeneration (for reviews, see Burda and Sofroniew 2014; Mosher and Wyss-Coray 2014; Ransohoff and Perry 2009).

Differences in diseases and reactive glia responses have important ramifications for targeting neuroinflammatory responses as an intervention strategy. Disease-modifying therapeutics that inhibit glial activation responses will need to be selective in their action, act at the appropriate stage of disease progression, and modulate endpoints or signaling pathways relevant to the particular neurodegenerative disease indication. There are numerous potential avenues for target-specific interventions in molecular events associated with neuroinflammation. The disease-modifying therapeutic could act by modulating the ability of the glia to perform a particular cellular function, such as releasing matrix metalloproteases to remodel the extracellular matrix. Alternatively, the therapeutic agent could block the ability of the glia to recognize the activating stimuli, such as blocking the response to the damage signal ATP by inhibiting purinergic receptor signaling. Another possibility for a disease-modifying therapeutic would be not to inhibit the glia response, but instead to block the ability of that response to elicit an effect on a target cell type such as the neuron. An example of this approach would be the use of a biologic, such as IL1ra, to block IL-1β dependent cytokine signaling.

Extensive evidence has implicated dysregulation and overproduction of proinflammatory cytokines as a contributor to pathophysiology progression in both chronic and acute neurodegenerative disorders. Taken in its entirety, the evidence is consistent with the hypothesis that proinflammatory cytokine up-regulation is a comparatively early event in the progression of pathophysiology that is causally linked to synaptic dysfunction, behavior deficits and, in the more extreme case, neuronal death (Van Eldik et al. 2007). This raises the possibility that blocking the production of specific proinflammatory cytokines, and/or blocking the receptor signaling events in neurons, for example, could be an effective strategy with potential for disease modification in multiple diseases and clinical presentations.

One of the most well established intracellular signal transduction cascades involved in the production of proinflammatory cytokines, and cytokine receptor signaling, in both peripheral and central inflammatory disorders, is the p38 MAPK family, especially the p38α isoform (Arthur and Ley 2013; Bachstetter and Van Eldik 2010). Using both a pharmacological approach with a small molecule p38α inhibitor and a genetic approach with primary microglia deficient in p38α, we previously showed (Bachstetter et al. 2011) that this isoform is critical for the production of cytokines from activated microglia. We also demonstrated (Xing et al. 2011, 2013) that microglial p38α-mediated cytokine overproduction is critical to inflammation-induced neurotoxicity, whereas the p38β isoform is not required for proflammatory cytokine production or neurotoxicity. Consistent with the above findings, we showed that myeloid-specific deletion of p38α protects mice from diffuse brain injury-induced vestibulomotor impairments and synaptic protein loss (Bachstetter et al. 2013). Overall, an accumulating body of knowledge demonstrates the importance of p38α MAPK as a central driver of pathological microglial activation and detrimental inflammatory responses, rendering the development of p38α MAPK inhibitors as an attractive avenue of exploration for new therapies against CNS diseases and injuries that involve injurious proinflammatory cytokine overproduction.

In addition to the role of p38α MAPK in glia, endogenous neuronal p38α MAPK can also play a role in pathology progression. For example, neuronal p38α MAPK has been implicated in the synaptic dysfunction brought about by excessive proinflammatory cytokine related neuroinflammation in diverse preclinical models (Tong et al. 2012). Similarly, axon-autonomous activation of p38α MAPK has been implicated in a gain of toxic function in SOD1 familial amyotrophic lateral sclerosis due to its role in the regulation of fast axonal transport (Morfini et al. 2013), which can contribute to the dying-back axonopathy found in such neurodegenerative disorders.

The prevailing evidence, therefore, raises the potential of a novel pharmacological paradigm in which enhanced responses might be possible via targeting the same molecular target in multiple cell types involved in CNS pathology progression (i.e. neurons and glia). The novel CNS paradigm stands in contrast to previous efforts at targeting p38 MAPK in peripheral tissue disorders where preclinical studies and clinical trials showed a pharmacodynamics effect of attenuating blood proinflammatory cytokine levels, but the direct linkage of the proinflammatory cytokine levels to end organ damage was less than ideal or engagement of target tissue p38 MAPK activity was not established.

The major challenge for p38 MAPK inhibitor in vivo studies in CNS disorders was, until recently, the issues found generally for targeting protein kinases in the CNS (Chico et al. 2009). The initial major challenge facing any CNS-targeted drug discovery program is effective blood–brain barrier (BBB) penetration, as only ~2 % of small molecule drugs exhibit adequate CNS exposure (Pardridge 2005). Another challenge in development of p38 MAPK inhibitors has been the ability to achieve selective inhibition of the kinase, as most of the extant inhibitors target multiple kinases in addition to p38. This early dependence on mixed kinase inhibitors for p38 MAPK in hundreds of publications has generated some confusion in the field of signaling research, as well as concern about the potential of off-target adverse events for drugs developed from these chemical scaffolds. Especially perplexing are conclusions based on the mixed p38 MAPK /casein kinase (CK) inhibitors. For example, re-investigation has revised conclusions about causative signaling pathways from being p38 MAPK mediated to being CK1 mediated (Shanware et al. 2009; Verkaar et al. 2011), and medical genetics outcomes forecast that decreased CK1 activity can be a susceptibility factor for migraine (Brennan et al. 2013). Therefore, it is critical that newly developed p38α MAPK inhibitors be extensively characterized for kinase selectivity using large-scale kinome screens, especially for CK crossover, and their biological mechanism of action be well-validated.

In this regard, we recently reported (Watterson et al. 2013) the development of a set of efficacious and CNS-penetrant small molecule p38α inhibitors that large-scale kinome screens show are selective for the p38 MAPK family, lack crossover to major GPCR agonist or antagonist classes based on functional screens, exhibit low toxicity at high doses, and are efficacious in an Alzheimer’s disease-relevant progressive brain injury model. The mechanism of pharmacological action, or pharmacodynamic effect, includes the suppression of stressor-induced increases in proinflammatory cytokine production. However, there remains a need for further exploration of biological target engagement and pharmacodynamic action if caveats from prior art are to be addressed fully.

We report here that the selective inhibitor MW181 engages its cellular p38α MAPK target with resultant attenuation of endogenous substrate phosphorylation in activated microglia cultures. We also report the dose-dependent linkage between the early decrease in substrate phosphorylation and the downstream attenuation of increases in proinflammatory cytokine levels. The extension to in vivo mechanisms is shown by the ability of orally administered MW181 to prevent stressor-induced increases in brain cytokines and chemokines in wild-type (WT) mice but not in genetically engineered p38α MAPK inhibitor-resistant mice. These results provide further validation of the utility of MW181 as a highly selective chemical biology tool appropriate to study the role of p38α MAPK in CNS disorders that have neuroinflammation as a component of the disease mechanism.

## Materials and Methods

### MW181

MW01-10-181SRM (=MW181) was synthesized as described (Watterson et al. 2013). MW181 is a water soluble, low molecular weight, acidic, small molecule. The HCl salt of MW181 was used for the cellular and in vivo studies described here. Stock solutions were made in sterile 0.9 % sodium chloride free of preservatives.

### Cell Culture

MW181 inhibition of lipopolysaccharide (LPS)-induced responses was tested in the murine microglial BV-2 cell line as previously described (Watterson et al. 2013). Briefly, cells were treated with either saline vehicle control or 100 ng/ml LPS (Salmonella enterica serotype typhimurium (Sigma-Aldrich: EU/mg of LPS is 600,000)), in the absence or presence of increasing concentrations of MW181. MW181 was added to the cultures immediately before the addition of LPS. Cells were harvested after 1 h of stimulation for western blot analysis and after 16 h of stimulation for proinflammatory cytokine measurements. Cell permeability and efflux pump susceptibility (permeability glycoprotein, Pgp) were determined by Apredica, Inc (Watertown, MA) using the standard Caco-2 two way permeability analysis (Stewart et al. 1995) in the absence and presence of a known Pgp inhibitor, verapamil, and monitoring parent drug by HPLC/MS/MS.

### Animals

All experiments were conducted in accordance with the Guide For the Care and Use of Laboratory Animals and approval of the Institutional Animal Care and Use Committee of the University of Kentucky. Animal experiments followed the recent NIH guidelines for rigor in study design and analysis (Landis et al. 2012; Shineman et al. 2011), including randomization of animals, and blinding of treatment groups and tissue samples. Male and female, 2- to 4-month-old C57Bl/6 mice (Harlan) were used as wild type (WT) mice. Aged-matched, male and female, p38α MAPK drug-resistant knock-in (p38α^T106M^) mice (p38α KI mice) were generated as previously described (O’Keefe et al. 2007). In vivo screening of MW181 compound efficacy was conducted as previously described (Watterson et al. 2013). Briefly, MW181 (5 or 20 mg/kg) or saline vehicle was administered by oral gavage (po) in a volume of 200 μL, and mice were given an intraperitoneal injection of saline vehicle or LPS (300,000 EU in 100 μL) 1 h later. Mice were euthanized at 6 h after LPS administration, based on our previous studies showing that this timepoint is optimal for IL-1β production. Brain was dissected on ice, snap-frozen in liquid nitrogen, and stored at −80 °C until time of use.

### Western Blotting

Western blotting analysis was performed as previously described (Bachstetter et al. 2011), using the following primary antibodies from Cell Signaling Technology (Beverly, MA): pMSK1 (cat. no. 9595 (1:1000)); pMK2 (cat. no. 3041 (1:1000)); GAPDH (cat no. 2118 (1:10000)).

### Cytokine/Chemokine Measurements

IL-1β in BV2 lysates was measured using kits from Meso Scale Discovery (MSD; Gaithersburg, Maryland), and cytokine/chemokine levels in mouse cortex lysates were measured using the MSD V-plex assay as previously described (Watterson et al. 2013).

### Statistics

**Fig. 1:**
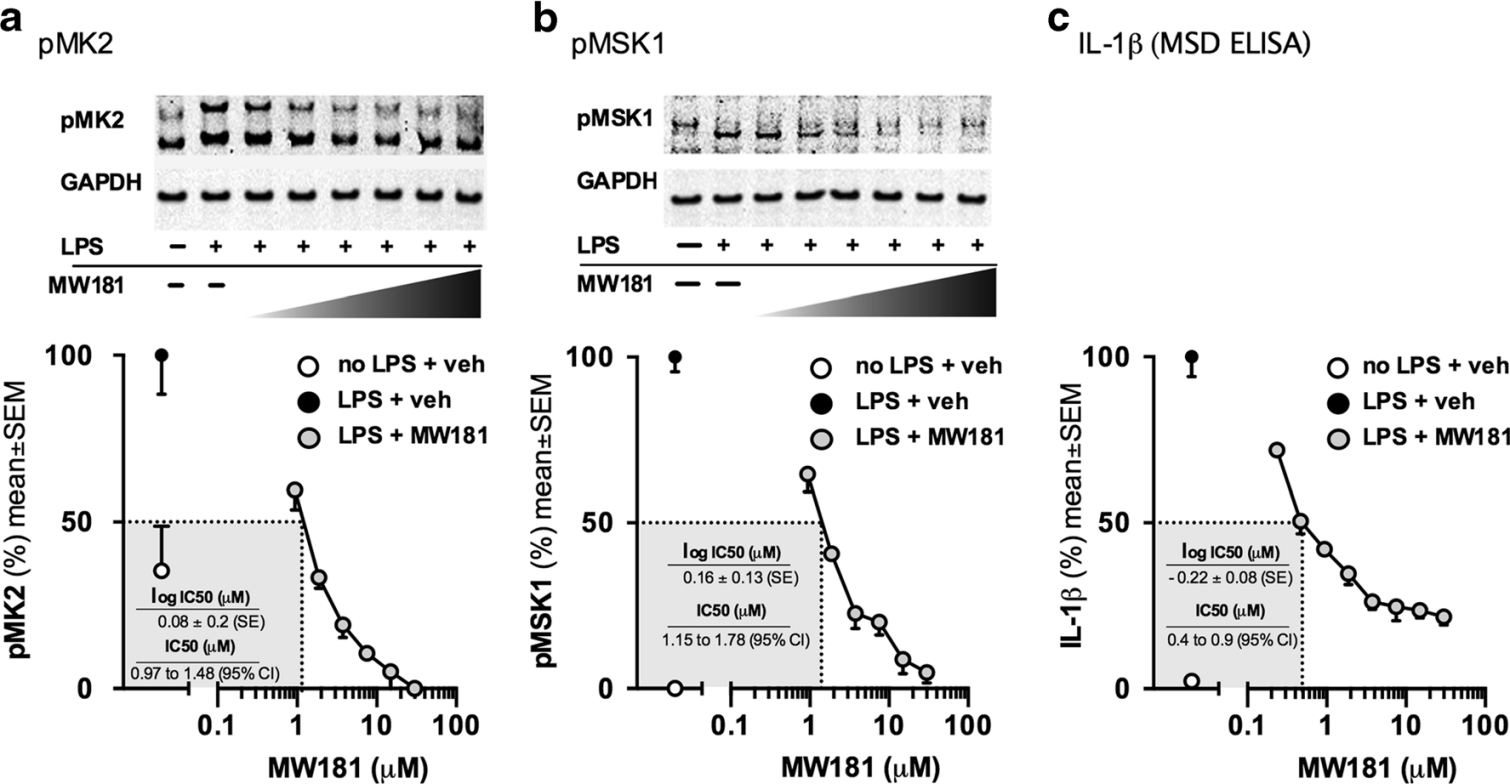
Concentration-dependent ability of MW181 to engage the endogenous glia target and suppress proinflammatory cytokine induction. Serial dilutions of the p38α MAPK inhibitor, MW181, starting at 30 μM, were added to BV2 cells stimulated with 100 ng/ml of LPS. The levels of phosphorylated (activated) p38α MAPK substrates pMK2 (**a**) and pMSK1 (**b**) were determined by western blot of cell lysates at 1 h after LPS addition. At 16 h after LPS addition, levels of the proinflammatory cytokine IL-1β (**c**) were measured in cell lysates by MSD ELISA. Data are presented as percent of maximal activity (activity with LPS + vehicle), and are representative of at least two independent experiments. The log IC_50_ values and the IC_50_ 95 % confidence intervals are shown in the gray boxes within the graphs

**Fig. 2:**
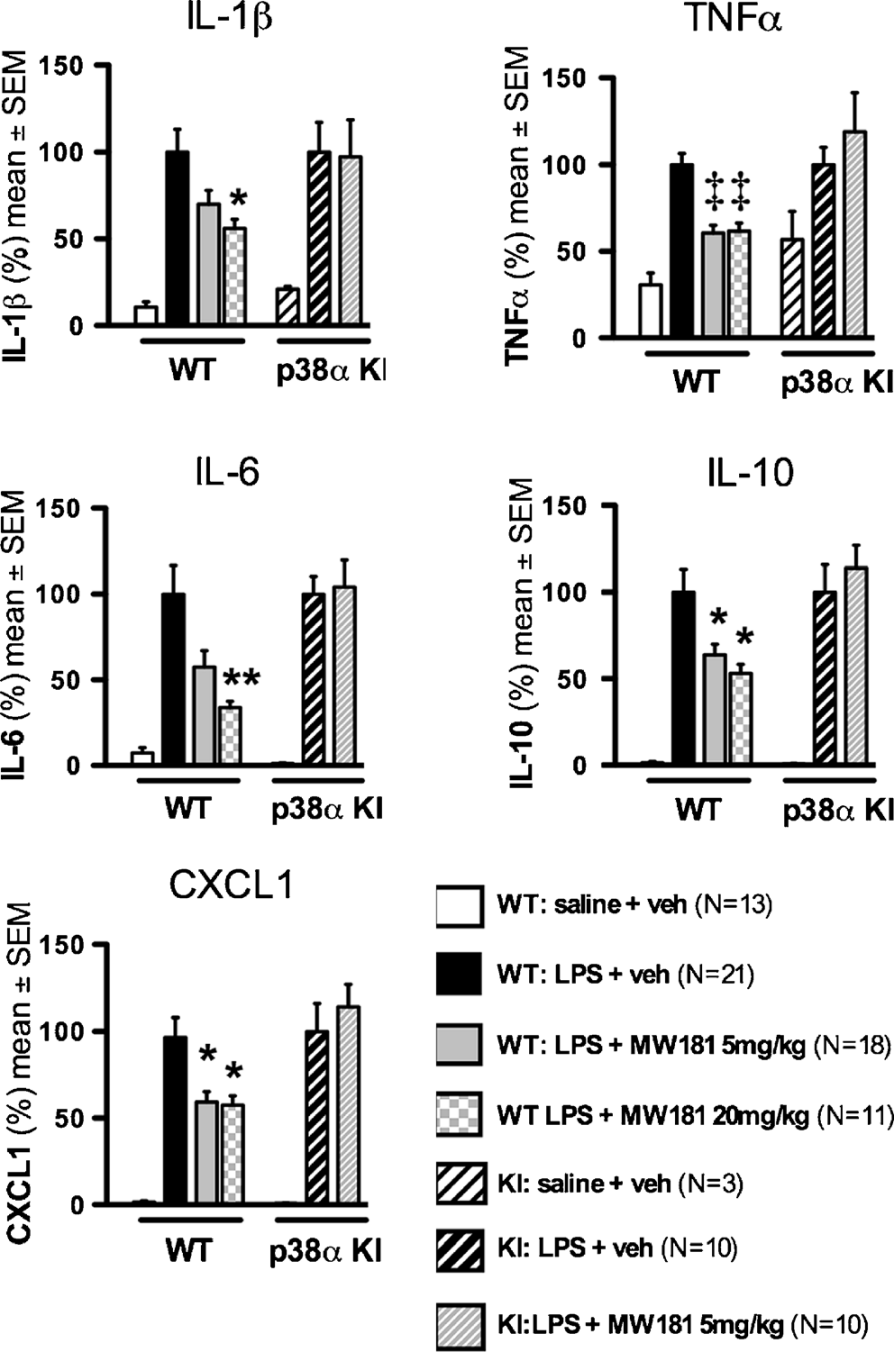
MW181 pharmacodynamic effect in WT mice and lack of effect in drug-resistant p38α^T106M^ MAPK KI mice. Mice were administered either saline vehicle or MW181 by oral gavage 1 h prior to an intraperitoneal LPS (300,000 EU) injection. Cortex was harvested at 6 h after LPS injection. Levels of inflammatory analytes in the cortical lysates were measured using MSD v-plex multiplex ELISAs. Data is a summary of four independent experiments. The experiments were normalized as a percent of the average of the LPS + veh for the respective experiments. Level of significance is denoted by *p < 0.05, **p < 0.01, and ‡*p* < 0.001

Statistical analysis used GraphPad prism software version 6 (GraphPad Software, San Diego California USA, www.graphpad.com). For Fig. 1, calculations of IC_50_ values were made using a nonlinear regression with a variable Hill slope, with the data normalized to the positive control to fit the top and bottom plateaus. For Fig. 2, effect of drug treatment in LPS-stimulated WT mice was analyzed using a one-way analysis of variance (ANOVA) followed by Tukey’s multiple comparison test. Reported P values are adjusted to account for multiple comparisons. Effect of MW181 treatment in p38α KI mice was analyzed using a two-tailed unpaired t-test. No statistical comparisons were made between mice treated with and without LPS, and no statistical comparisons were made between WT and p38α KI mice. Therefore, to conserve on the total number of mice needed, the number of mice used in the no LPS p38α KI group was limited to *n* = 3. Significance was defined as a *p* < 0.05. Unless otherwise indicated, values are expressed as mean ± SEM.

## Results

### MW181 is Freely Cell Permeable and not a Substrate for the Pgp Efflux Pump

Interpretation of cellular mechanism of action studies for small molecules requires adequate cell membrane permeability and minimal removal by efflux pumps during the course of the experiment. A drug predominantly moves by passive diffusion through cellular membranes (A→B direction) that comprise tissue barriers. If the drug is a PgP substrate, it is pumped back out (B→A direction) and eventually excreted from the body. The Pgp efflux pump status is especially important for CNS studies, as the pump removes susceptible small molecules from cells and is a major contributor to blood brain permeability problems for the vast majority of small molecule drugs and inhibitors. In addition, Pgp status can change with disease, drug treatment or innate immune status. The caco-2 bi-directional transport assay is a calibrated standard approach used to address permeability, which is a reflection of the drug’s molecular properties, and to determine if the drug is a PgP substrate. Briefly, cells are grown as a monolayer in a transwell, allowing directional measurement of transport. The net flux is determined as a ratio of B→A/A→B. If the ratio is >2, then more of the drug is pumped back out than enters by passive diffusion, usually due to the drug being a PgP substrate. Status is confirmed by incubating in the presence of a saturating concentration of a specific PgP substrate, verapamil. The competition for the PgP transporter results in the efflux ratio for the drug changing from >2 to <2. As summarized in Table 1, MW181 exhibits efflux ratios <2 in both the absence and presence of a specific PgP inhibitor, demonstrating that MW181 has high permeability and is not a Pgp efflux pump substrate.

**Table 1:** MW181 has high cell permeability and is not a substrate for Pgp efflux pump. For apical to basolateral (A→B) permeability, test agent added to apical side and permeation determined by LC/MS/MS measured on basolateral side; the opposite is done for B→A. Pgp substrate status determined by effect of standard inhibitor verapamil on bidirectional flux

Sample	Test concentration (μM)	Assay duration (h)	Mean A→B ^a^P_app_ (10^−6^ cm s^−1^)	Mean B→A ^a^P_app_ (10^−6^ cm s^−1^)	^b^Efflux ratio	Recovery	Notes
MW181	10	2	36.7	42.5	1.2	88 %	High permeability
MW181 + verapamil	10	2	45.2	42.9	0.9	98 %	Not a Pgp substrate

^a^Apparent permeability

^b^P_app_ (B→A)/ P_app_ (A→B)

### MW181 Engages Endogenous Glia p38α MAPK in a Concentration-Dependent Manner

Mitogen-activated protein kinase-activated protein kinase 2 (MK2) and mitogen- and stress-activated kinase 1 (MSK1) are downstream substrates of p38α MAPK. To assess target engagement by MW181, we measured the levels of phosphorylated (p)-MK2 and pMSK1 in LPS-stimulated glia treated with MW181 at different concentrations. In our culture conditions without LPS, a basal activation of pMK2 is observed, which is further enhanced by LPS stimulation. MW181 was able to suppress both basal and LPS-induced levels of pMK2 with an IC_50_ of 1,197 nM (Fig. 1a). MW181 also inhibited LPS-stimulated pMSK1 levels, with an IC_50_ of 1,430 nM (Fig. 1b).

### MW181 Attenuates Stressor-Induced Proinflammatory Cytokine Up-Regulation in Microglia

LPS-induced changes in proinflammatory cytokine levels are evident at later time points compared to the changes in endogenous substrate phosphorylation, consistent with prevailing models of the signal transduction mechanism. We, therefore, tested the ability of MW181 to suppress LPS-induced up-regulation of the proinflammatory cytokine IL-1β in the BV2 microglia cell line at 16 h vs 1 h for substrate phosphorylation. MW181 inhibited IL-1β production in a concentration-dependent manner, with an IC_50_ of 599 nM (Fig. 1c).

### Orally Administered MW181 Shows Dose-Dependent Inhibition of CNS Cytokine Up-Regulation

Replicating the IL-1β result in vivo and extending the data of Watterson et al. (2013), we found (Fig. 2) a significant effect of MW181 on cortical levels of IL-1β in wild type mice (F_(2, 47)_ = 4.041; *p* = 0.024). MW181 at 20 mg/kg suppressed IL-1β levels in LPS-challenged mice (*p* = 0.031), and at 5 mg/kg showed a trend, but when corrected for multiple comparisons the decrease did not reach significance. MW181 showed a strong inhibition of TNFα up-regulation (F_(2, 47)_ = 16.39; *p* < 0.0001), with both the 5 mg/kg and 20 mg/kg doses significantly suppressing TNFα levels (*p* < 0.0001, and *p* = 0.0002; respectively). There was also a significant effect of MW181 on IL-6 levels in LPS-treated WT mice (F_(2, 47)_ = 5,808; *p* = 0.0056); again, 20 mg/kg of MW181 inhibited IL-6 levels (*p* = 0.0071), but the inhibition by MW181 at 5 mg/kg did not quite reach significance (*p* = 0.055). At this time window, MW181 also decreased the anti-inflammatory cytokine IL-10 (F_(2, 47)_ = 5.436; *p* = 0.0075) at both compound concentrations (5 mg/kg, *p* = 0.0334; 20 mg/kg, *p* = 0.0152). However, under the conditions of this study it cannot be stated if this is a direct effect of MW181 or feedback loop responses to blockage of proinflammatory cytokine increases. The cortical levels of CXCL1, a chemokine involved in neutrophil and oligodendrocyte precursor cell recruitment (Ransohoff 2009), were also reduced by MW181 treatment (F_(2, 47)_ = 5.834; *p* = 0.0055). Both the 5 mg/kg and 20 mg/kg doses of MW181 significantly suppressed CXCL1 levels compared to the LPS-stimulated WT mice treated with vehicle (*p* = 0.0122, and *p* = 0.025; respectively).

### Lack of MW181 Pharmacodynamic Effect in the p38α^T106M^ MAPK Drug-Resistant KI Mice

To further assess in vivo target specificity of MW181 and better link kinase target engagement by the inhibitor to in vivo pharmacodynamic endpoints, we took advantage of the p38α^T106M^ KI mice (O’Keefe et al. 2007). These KI mice have the endogenous p38α MAPK gene disrupted, but produce a functional protein kinase resistant to inhibitors that bind in an active site hydrophobic pocket. The molecular mechanism of drug resistance is the targeted replacement of the gatekeeper Thr at residue 106 with a larger side chain Met amino acid that renders the hydrophobic pocket less accessible to inhibitors with bulky substituents that occupy this pocket. We previously showed through the combined use of high-resolution co-crystallography and comparative structure-activity relationships of MW181 analogs (Watterson et al. 2013) that MW181 has a naphthyl substituent that occupies this pocket in p38α MAPK. This substituent contributes to the drug:target affinity as well as to the selectivity of MW181 for p38α MAPK vs other proteome targets that lack the three dimensional features required to engage MW181 in high affinity binding. Therefore, this drug-resistant mutant mouse model is an excellent tool to explore directly any potential off-target effects of MW181 relevant to the pharmacodynamic effect on proinflammatory cytokine production. In LPS-stressed p38α KI mice, MW181 was unable to suppress any of the neuroinflammatory mediators (IL-1β, TNFα, IL-6, IL-10, or CXCL1), showing levels comparable to the LPS-stimulated p38α KI mice treated with saline vehicle (Fig. 2).

## Discussion

The studies reported here demonstrate that MW181 engages the p38α MAPK target in its glia cellular context and links the target engagement to the downstream pharmacodynamic effect of normalized CNS proinflammatory cytokine levels. The in vivo selectivity of MW181 action is further indicated by the failure to bring about its pharmacodynamic effects in p38α MAPK inhibitor resistant mice. Taken in its entirety, the results presented here and previously (Bachstetter et al. 2011; Xing et al. 2013; Watterson et al. 2013) strongly indicate that the pharmacodynamic effects of this class of novel, highly selective inhibitors of CNS p38α MAPK are via modulation of endogenous p38α MAPK and not other protein kinases, or serendipitous off-target mechanisms. Our results further document the utility of MW181 to probe the role of p38α MAPK in disorders of the CNS that include a neuroinflammatory component as part of the disease mechanism.
